# Controllability in Cancer Metabolic Networks According to Drug Targets as Driver Nodes

**DOI:** 10.1371/journal.pone.0079397

**Published:** 2013-11-25

**Authors:** Yazdan Asgari, Ali Salehzadeh-Yazdi, Falk Schreiber, Ali Masoudi-Nejad

**Affiliations:** 1 Laboratory of Systems Biology and Bioinformatics (LBB), Institute of Biochemistry and Biophysics, University of Tehran, Tehran, Iran; 2 Leibniz Institute of Plant Genetics and Crop Plant Research Gatersleben, Gatersleben, Germany; 3 Institute of Computer Science, Martin Luther University Halle-Wittenberg, Halle, Germany; 4 Clayton School of Information Technology, Monash University, Clayton, VIC, Australia; Semmelweis University, Hungary

## Abstract

Networks are employed to represent many nonlinear complex systems in the real world. The topological aspects and relationships between the structure and function of biological networks have been widely studied in the past few decades. However dynamic and control features of complex networks have not been widely researched, in comparison to topological network features. In this study, we explore the relationship between network controllability, topological parameters, and network medicine (metabolic drug targets). Considering the assumption that targets of approved anticancer metabolic drugs are driver nodes (which control cancer metabolic networks), we have applied topological analysis to genome-scale metabolic models of 15 normal and corresponding cancer cell types. The results show that besides primary network parameters, more complex network metrics such as motifs and clusters may also be appropriate for controlling the systems providing the controllability relationship between topological parameters and drug targets. Consequently, this study reveals the possibilities of following a set of driver nodes in network clusters instead of considering them individually according to their centralities. This outcome suggests considering distributed control systems instead of nodal control for cancer metabolic networks, leading to a new strategy in the field of network medicine.

## Introduction

Ever since Otto Warburg discovered the unique characteristics of tumor cell metabolism over 80 years ago [1], the interpretation of cancer as a genetic disease has gradually been displaced by the understanding of it as a metabolic disease [2]. Cancerous cells have to reprogram their metabolic states during tumor initiation and progression through genetic and epigenetic alterations in metabolic genes, in order to respond to the demanding requirements for growth [3]. Understanding the details of human metabolism has facilitated the reconstruction of genome-scale metabolic models (GEMs) of various cell types and diseases. [4]–[6]. There are four generic reconstructed genome-scale human metabolic networks: Recon1 [7], Recon2 [8], the Edinburgh Human Metabolic Network (EHMN) [9], and HumanCyc [10]. For the study of particular human cell types, tissue-specificity, and cancer; metabolic models have been reconstructed either manually or automatically. Manually reconstructed metabolic models include models of the liver (HepatoNet1, [11]), kidney [12], brain [13], erythrocytes [14], alveolar macrophages [15] as well a model of the core metabolic pathways participating in cancer growth [16]. The first automatic reconstructed metabolic model has been developed by Schlomi et al. for 10 different human tissues [17] as subsets of Recon1. Later they proposed a different algorithm to generate a more flexible and functional tissue-specific model [18]. Folger et al. [19] have constructed a large-scale metabolic model of different cancers. Agren et al. [20] have developed the INIT algorithm (Integrative Network Inference for Tissues) which relies on the Human Protein Atlas (HPA) as the main evidence source, and on tissue-specific gene expression data [21] and metabolomic data from the Human Metabolome DataBase (HMDB) [22] as extra sources of evidence. Finally, Wang et al. [23] have developed a new approach named metabolic Context-specificity Assessed by Deterministic Reaction Evaluation (mCADRE) in order to build 126 human tissue-specific metabolic models.

Reconstructed human metabolic networks provide a useful tool for the study of diseases and the development of drugs. Several simulations and modeling methods have been developed to address the issues of drug-target prediction [24]–[28]. The topological features of metabolic networks contribute to the robustness and flexibility of the complex biosystems and may explain, in general, the fact that many drug candidates are ineffective (the drug effect is compensated by other pathways in the network) or show unexpected severe side effects [29]–[31]. Prompted by these findings, many scientists have proposed a system-oriented drug design strategy to replace the current “one gene, one drug, one target, one disease” approach [31]–[33]. Hence the concept of polypharmacology has been proposed for those drugs acting on multiple targets instead on one target [34]. It is also reasonable that multiple target modifications can more effectively convert the system from a disease state to a normal state than a single target modification. In fact, successful applications of multi-component therapies have been reported and multi-component drugs are already on the market [35], [36]. Systems analysis will help us not only in the discovery of novel drug targets but also in developing new systems-based therapy strategies [37].

Network medicine is a new subject that tries to link topological network properties to biological function and disease. Network medicine explores the molecular complexity of a special disease and relationships between distinct phenotypes which may lead to the identification of disease modules and pathways [38]. A better understanding of the implications of cellular interconnectedness for disease progression will lead to discovery of new disease genes and pathways. These advances may also reshape clinical practice, from discovery of more accurate biomarkers to a better disease classification leading to personalized therapies and treatment. Recently, there have been some studies on disease clustering approaches which aim to find different disease modules and predict new genes. Barabasi et al. [39] have shown that each disease has its own unique module and that different disease modules can overlap. In another study with respect to the prediction of new genes, Chen et al. [40] have validated three unknown genes (LPL, LACTB, and PPM1L) as obesity genes in transgenic mice. In other work, Oti et al. [41] have found Janus kinase 3 (JAK3) as a candidate protein for severe combined immunodeficiency syndrome. The controllability of networks is becoming a key issue in many disciplines, including sociology and biology [42]–[45]. Network controllability is the ability to guide a system's behavior towards a desired state through appropriate management of some input variables [46], [47]. The difficulty in control theory is because of the system's architecture and the dynamical rules which makes controllability to be possible only in systems where both issues are well mapped [48]. In the last decades, it has been demonstrated it is fair to expect that the network topology would definitely affect controllability as well. This approach helps us avoid any entanglement due to nonlinear effects and consideration of networks with thousands to millions of nodes [49]. So, structural controllability could be an appropriate choice for dealing with large biological networks. Despite extensive interest in the study of topological features over the last decade, dynamic and control aspects of complex networks have not followed the same pace of research development. For example, different topological properties such as highly connected nodes, betweenness and closeness centralities have been chosen as candidates for an encoding part of system controllability but there is no agreement at present on what network property is suitable for [48], [50], [51]. Liu et al. [48] have proposed analytical tools for the controllability of complex networks. Their approach is based on the identification of a subset of nodes (called driver nodes) in a directed network that can control the dynamics of the system. They have shown that the number of driver nodes is determined primarily by the degree distribution of a network. It means that while homogeneous (dense) networks could be controlled using a few driver nodes, inhomogeneous (sparse) networks (found in many real networks) are the most difficult to control due to a high number of driver nodes. In addition, driver nodes tend to avoid high-degree nodes (called hubs) in both dense and sparse (real) systems. Consequently, random networks are easier to control [48]. One year later, they have introduced a new network centrality called control centrality in order to address the importance of a given node in maintaining a system's controllability [51].

There have been reactions to Liu's work. Ferrarini [52] has introduced five unconventional thoughts on Liu's approach using the control of edges instead of nodes, which may be more useful in complex networks. In another comment, Benarjee et al. [53] have doubts about using degree centrality for controlling a system. They believe that an effective understanding of controllability in directed networks can be reached using distance based measures of closeness centrality (CC) and betweenness centrality (BC), and may not require the knowledge of local connectivity measures such as in-degree and out-degree, because degree reflects information about the immediate neighborhood of a node. In contrast, CC and BC signifies a node's potential to communicate with further nodes through the network. This shows the important role that CC and BC may play in determining controllability. However the main challenge still remains to determine which node is the driver node. In 2012, Nepusz et al. considered the controllability of a system based on edge dynamics. In this approach, each node accepts information through its inbound edge and spreads the results to its neighbouring nodes using the outbound edges [54]. They have shown that networks with scale-free degree distribution are easier to control. In the same year, Nacher et al. [50] introduced a new approach which investigated the dependence of the size of the minimum dominating set (MDS) of nodes on topological features of directed real networks for the purposes of control design. Having computed the MDS in real networks and in computer-generated networks with a variety of topologies, they demonstrated that the MDS size depends on the average degree of all nodes in the network. They have shown that the more homogeneous a network, the larger the fraction of individuals required for dominating the entire system. Also, the more heterogenous a network is, the easier it is to control the system. In addition, the MDS tends to target highly connected nodes, which is in contrast to the Liu et al. study [48]. However they mention that their results do not contradict Liu's work because of different strategies. Liu's work assumes that only driver node values could be directly controlled through external signals, whereas the MDS method undertakes that each driver node is sufficiently smart to control individual links separately. On the other hand, in the MDS approach, a node with degree *k* is treated as if it were a set of *k* nodes [50]. So they believe that the MDS approach complements Liu's results.

Both nodal and edge dynamics frameworks (the approaches mentioned above) have been implied for covering unipartite graphs. In 2013, Nacher et al. introduced a modified version of MDS in order to study the controllability of bipartite networks. The results demonstrated that MDS tends to select high degree nodes and nodes with a high betweenness centrality in bipartite networks. But the author mentioned that this approach may not be possible in some kinds of biological networks such as Protein-Protein Interaction (PPI) and metabolic networks.

In this paper, we have tried to explore possible relation between topological analysis, structural controllability, and metabolic networks. We have applied a comprehensive (local and global) topological analysis of recently published genome-scale metabolic models of normal and cancer tissue-specific models to assess the controllability relation between topological parameters and drug targets (as driver nodes),with the assumption that the targets of approved anticancer metabolic drugs are driver nodes and therefore control cancer metabolic networks. In addition, the outcomes of metabolic networks controllability could create insights leading to the discovery of novel drug targets. We have shown that besides primary network parameters, more complex network metrics such as motif and clusters may also provide new tools for addressing network controllability in metabolic networks. Characterizing the drug target in enzyme-centric clusters shows that most of the drug targets belong to one specific cluster of an enzyme-centric network. This could provide new insight into considering distributed control systems (DCS) instead of nodal control for cancer metabolic networks [55], [56]. If so, DCS may be considered as a new strategy in the field of network medicine.

## Materials and Methods

### Metabolic and enzyme-centric network construction of cancer and normal cell types

Omics data such as transcriptomic data are often noisy. In addition, mRNA expressions are relative to a reference condition and sometimes do not correlate well with enzyme levels [57]. Therefore, a prerequisite for reconstructing reasonable and reliable tissue-specific models is to consider other resources as well. We used metabolic networks of 15 cancer cell types and their corresponding normal cell types in our study (categorized according to Table 1). These networks have been constructed on the basis of the INIT algorithm [20] which integrates tissue-specific gene expression data into a general human metabolic model. Therefore, each normal and cancer model contains metabolites and reactions different to the others. We have written MATLAB scripts to compare metabolites and reactions between normal and cancer models (File_S1). Full lists of metabolites and reactions of all 15 cancers and their associated normal models are summarized in the Files_S2, S3.

**Table 1:** List of 15 cancer cell types and their corresponding normal cell types.

Cancerous cell	Normal cell
Breast	Breast Glandular
Cervical	Cervix squamous
Colorectal	Colon Glandular
Endometrial	Corpus Endometrial-Corpus Glandular
Renal	Kidney Glomeruli-Kidney Tubules
Liver	Liver Hepatocyt
Lung	Lung Alveolar
Ovarian	Ovary Stromal
Pancreatic	Pancreas Islet
Prostate	Prostate Glandualr
Skin	Skin Epidermal
Stomach	Stomach Glandular (I&II)
Testis	Testis Leydig
Thyroid	Thyroid Glandular
Urothelial	Urinary Bladder

All original SBML files have been downloaded from http://www.metabolicatlas.com/ [20].

**Figure 1:**
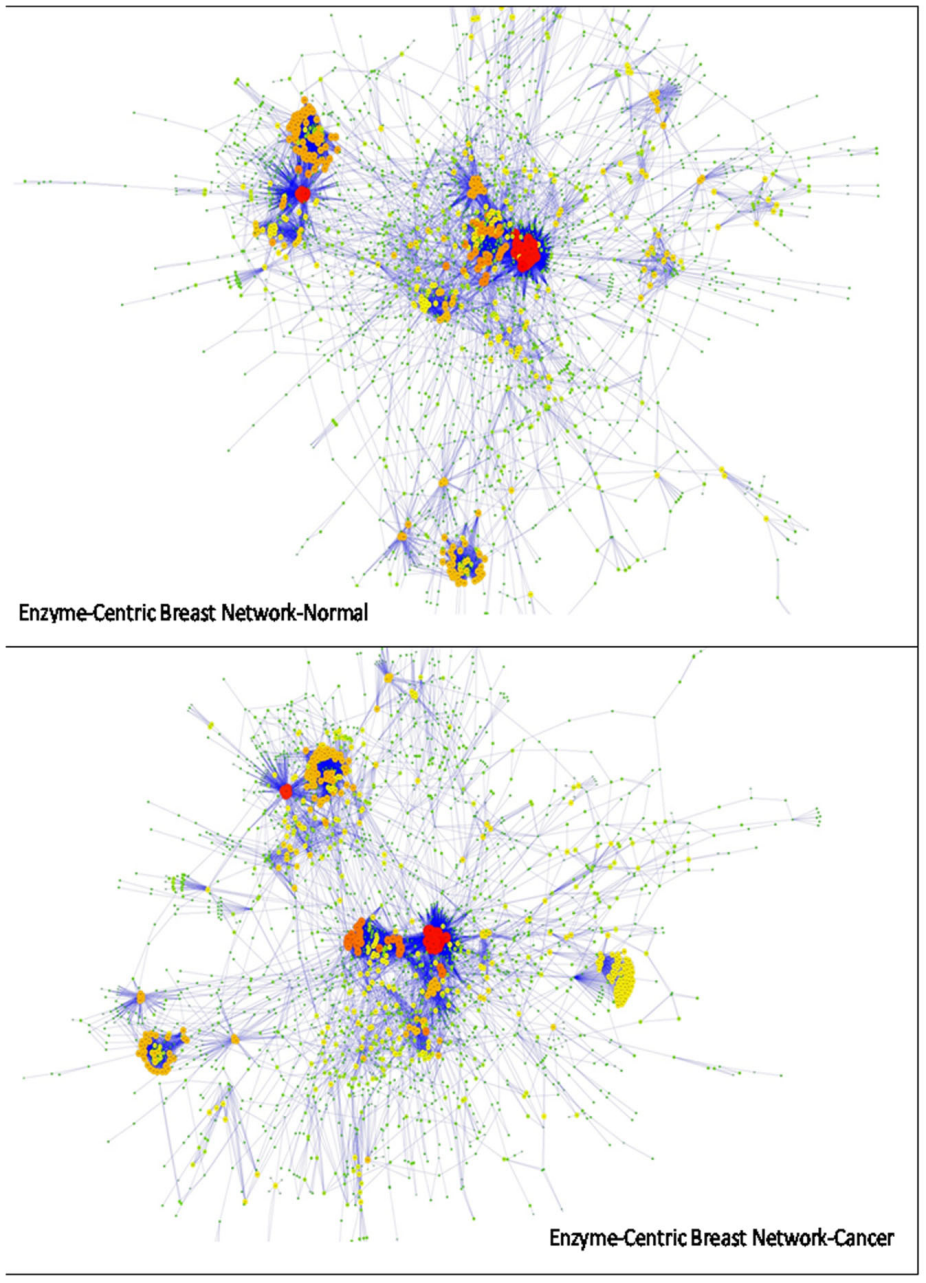
Directed enzyme-centric metabolic networks of cancer and normal breast cells.

The SBML files are bipartite graphs including two types of nodes (metabolites and reactions). Bipartite characteristics of metabolic networks make it difficult to analyse them with structural methods. In addition, metabolite-metabolite (metabolite-centric) and enzyme-enzyme (enzyme-centric) networks can provide extra insights and are therefore relevant for further analysis of the metabolism. It is also necessary to address structural controllability based on nodal dynamics (the approach of this study), and to construct metabolite- and enzyme-centric networks. We have written scripts in MATLAB software (R2012b) in order to construct undirected and directed metabolite-centric, as well as undirected and directed enzyme-centric networks based on SBML files. We have added network construction procedures including the algorithms in the File_S13. All the networks constructed are available in the File_S4. An example of a directed enzyme-centric metabolic network of cancer and normal breast cells imported in Cytoscape software [58] is shown in Figure 1. A summary of the kind of networks, the software and the parameters used for each analysis has been provided in Table 2.

**Table 2:** A summary of the different networks, software and parameters used for each topological analysis.

**Primary topological analysis**			
**Purpose of study:** to check any structural differences between metabolite- and enzyme-centric of normal and cancer networks			
**Networks Types**		metabolite-centric network	directed
			undirected
		enzyme-centric network	directed
			undirected
**Software**		*Network Analysi*s plugin in Cytoscape	
**Parameters**	**directed**	in-degree, out-degree, connected components, average number of neighbors, number of nodes, isolated node	
	**undirected**	degree, connected components, network diameter, network centralization, characteristic path length, average number of neighbors, total number of nodes, network heterogeneity, isolated node	
**Centrality analysis**			
**Purpose of study:** to check distribution of drug targets among the 100 top of centralities			
**Networks Types**		enzyme-centric network	directed
**Software**		*cytoHubba* plugin in Cytoscape	
**Parameters**		Maximal Clique Centrality (MCC), Density of Maximum Neighborhood Component (DMNC), Maximum Neighborhood Component (MNC), Degree, Edge Percolated Component (EPC), Bottleneck, Eccentricity, Closeness, Radiability, Betweenness, Stress, Clustering Coefficient	
**Motif discovery**			
**Purpose of study:** to check any differences between metabolite- and enzyme-centric of normal and cancer networks.			
**Networks Types**		metabolite-centric network	directed
		enzyme-centric network	directed
**Software**		*Quatexelero* algorithm	
**Parameters**		Motif of size 3 (13 types)	
**Clustering**			
**Purpose of study: (1)** to check any differences between number of clusters in normal and cancer networks, **(2)** to check distribution of drug targets among clusters			
**Networks Types**		enzyme-centric network	directed
**Software**		*MCODE* plugin in Cytoscape	
**Parameters**		degree cutoff = 2, without loops, node score cutoff = 0.2, K-core = 2, Max. Depth = 100, Include haircut, without fluff	

### Primary topological analysis of four different kinds of networks

Primary topological analysis has been carried out on four different networks of normal and cancer cell metabolic networks (undirected and directed metabolite-centric network, undirected and directed enzyme-centric network) using the *Network Analysi*s plugin in Cytoscape [58]. The in-degree, out-degree, connected components, average number of neighbors, number of nodes and isolated node parameters have been measured for direct networks. The degree, connected components, network diameter, network centralization, characteristic path length, average number of neighbors, total number of nodes, network heterogeneity and isolated node parameters have been measured for undirected networks. A summary definition of the different parameters is available in the File_S5. We have provided all power-law plots for every constructed network with fitting results in the File_S12.

### Centrality analysis

Centrality analysis has been carried out on the directed enzyme-centric networks of cancer and normal cell types using the *cytoHubba* plugin [59] in Cytoscape. We have used twelve centrality parameters: Maximal Clique Centrality (MCC), Density of Maximum Neighborhood Component (DMNC), Maximum Neighborhood Component (MNC), Degree, Edge Percolated Component (EPC), Bottleneck, Eccentricity, Closeness, Radiability, Betweenness, Stress and Clustering Coefficient [59].

### Motif discovery

**Figure 2:**
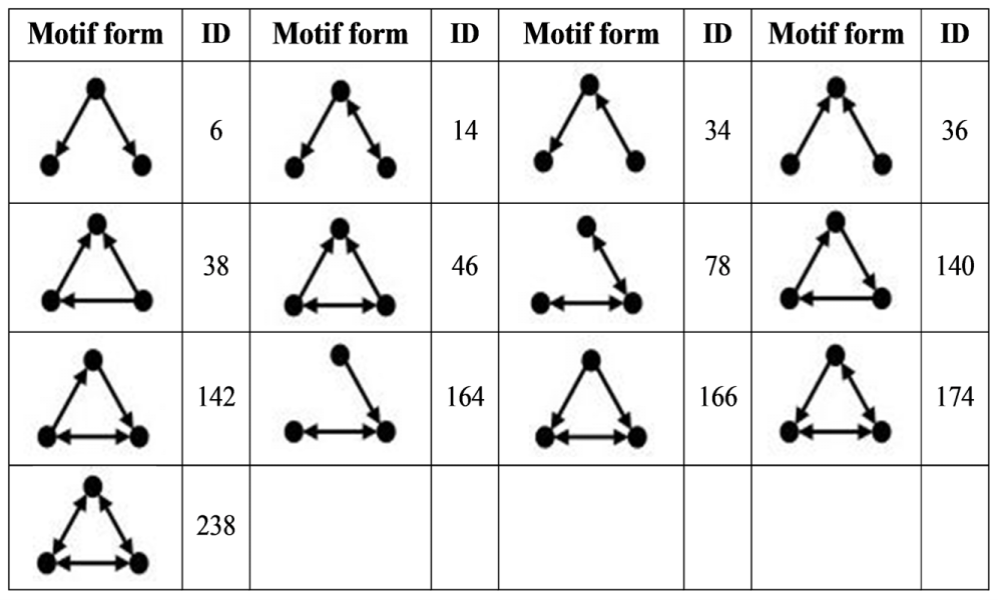
Corresponding Motif IDs of size 3 used in this study.

Motif finding has been carried out on the directed metabolic and enzyme-centric networks of cancer and normal cell types using the Quatexelero algorithm [60] (an enhanced Kavosh algorithm [61]). The analysis has been performed on motifs of size 3 (including 13 different types-Figure 2) because the motif of this size has been served as the building blocks of biological networks from bacteria to mammals [62].

### Clustering

Clustering analysis has been performed on the directed enzyme-centric networks of cancer and normal cell types using the *MCODE* [63] plugin in Cytoscape. Clustering parameters during analysis have been shown in Table 2.

### Anticancer metabolic drugs and their targets

For finding anticancer metabolic drugs and their targets, we have used the drug bank database [64]. All anticancer metabolic drugs and their targets are listed in the File_S6. The metabolic functions of the drug targets are listed in the File_S7. These data have been used for centrality and clustering analysis of enzyme-centric networks of cancer cell types.

## Results

### Primary topological analysis

Since metabolic networks satisfy power-law degree distribution, scale-free, and small-world properties [65], we have checked all constructed networks for basic network properties. Degree distribution of a scale-free network having *k* connections to other nodes satisfies the following relation [65]

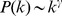
where 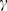 is power-law parameter. For all constructed networks, we have applied curve fitting to aforementioned relation and have calculated values of 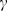 and 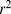 (coefficient of determination or R-squared). Our results show that 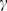 related to degree distribution (in-degree and out-degree for directed networks) for all metabolite-centric and enzyme-centric networks are less than two. According to the 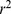, all networks are scale free. In addition, calculated characteristic path length values have been implied on small-world property. Clustering coefficient, network diameter and connected components are other topological parameters that relate to network heterogeneity. The primary topological parameters related to directed metabolite-centric networks are shown in Table 3. Complete lists of data are available in the File_S8. All power-law plots for every constructed network with fitting results are available in the File_S12.

**Table 3:** Primary topological measures related to directed metabolite-centric networks.

Name	In-degree		Out-degree		Clustering Coefficient	connected components	network diameter	characteristic path length	Avg. number of neighbors	number of nodes	isolated nodes	multi-edge node pairs
	*r* ^2^	*γ*	*r* ^2^	*γ*								
**Breast Cancer**	0.751	−1.381	0.775	−1.349	0.065	1751	15	5.23	3.582	4473	1746	466
**BreastGlandular**	0.736	−1.35	0.775	−1.343	0.074	1541	16	5.281	3.875	4313	1533	502
**Cervical Cancer**	0.748	−1.332	0.739	−1.315	0.075	1615	15	5.144	3.888	4443	1611	517
**Cervix Squamous**	0.716	−1.348	0.749	−1.326	0.065	1564	17	5.323	3.501	3900	1558	383
**Colon Glandular**	0.772	−1.347	0.763	−1.319	0.077	1742	16	5.192	3.91	4669	1735	594
**Colorectal Cancer**	0.761	−1.374	0.784	−1.353	0.069	1756	17	5.215	3.609	4469	1750	496
**Copus Endometrial**	0.728	−1.405	0.78	−1.394	0.056	1293	15	5.076	3.467	3329	1284	337
**Copus Glandular**	0.774	−1.389	0.77	−1.353	0.064	1720	17	5.298	3.495	4274	1716	377
**Endometrial Cancer**	0.739	−1.395	0.739	−1.343	0.069	1723	16	5.36	3.644	4389	1713	465
**Kidney Glomeruli**	0.766	−1.361	0.802	−1.352	0.069	1283	15	5.289	3.933	3798	1278	410
**Kidney Tubules**	0.757	−1.354	0.745	−1.303	0.063	1850	16	5.333	3.515	4599	1842	434
**Liver Cancer**	0.76	−1.378	0.774	−1.31	0.067	1796	16	5.298	3.644	4601	1788	493
**Liver Hepatocytes**	0.774	−1.35	0.779	−1.323	0.073	1715	17	5.418	3.885	4650	1707	536
**Lung Alveolar**	0.734	−1.348	0.761	−1.322	0.071	1447	14	5.078	3.914	4128	1437	488
**Lung Cancer**	0.742	−1.362	0.739	−1.348	0.07	1496	16	5.211	3.893	4267	1490	455
**Ovarian Cancer**	0.75	−1.359	0.773	−1.336	0.075	1625	14	5.249	3.856	4439	1620	523
**Ovary Stromal**	0.744	−1.36	0.77	−1.339	0.062	1293	14	5.038	3.617	3504	1286	334
**Pancreas Islet**	0.749	−1.347	0.749	−1.331	0.069	1662	15	5.079	3.688	4359	1652	480
**Pancreatic Cancer**	0.764	−1.382	0.727	−1.314	0.075	1621	16	5.227	3.899	4492	1613	521
**Prostate Cancer**	0.73	−1.363	0.775	−1.332	0.076	1567	16	5.182	3.923	4363	1560	492
**Prostate Glandular**	0.75	−1.368	0.756	−1.309	0.075	1561	14	5.175	3.936	4473	1557	485
**Renal Cancer**	0.772	−1.39	0.743	−1.323	0.076	1454	15	5.111	3.981	4306	1448	501
**Skin Cancer**	0.732	−1.363	0.739	−1.343	0.074	1393	15	5.193	3.971	4075	1386	479
**Skin Epidermal**	0.752	−1.348	0.764	−1.298	0.075	1444	16	5.115	3.9	4155	1434	459
**Stomach 1Glandular**	0.718	−1.337	0.798	−1.379	0.073	1617	14	5.088	3.906	4536	1610	495
**Stomach 2Glandular**	0.75	−1.363	0.769	−1.328	0.064	1824	14	5.19	3.536	4517	1816	421
**Stomach Cancer**	0.752	−1.36	0.784	−1.368	0.074	1518	14	5.217	3.893	4302	1511	497
**Testis Cancer**	0.756	−1.355	0.728	−1.372	0.072	1490	17	5.152	3.872	4148	1483	472
**Testis Leydig**	0.751	−1.342	0.757	−1.302	0.073	1776	15	5.193	3.802	4674	1768	519
**Thyroid Cancer**	0.752	−1.368	0.781	−1.338	0.073	1718	16	5.237	3.85	4673	1710	553
**Thyroid Glandular**	0.727	−1.348	0.725	−1.324	0.074	1771	16	5.241	3.837	4716	1762	525
**Urinary Bladder**	0.78	−1.386	0.725	−1.329	0.061	1795	15	5.252	3.476	4405	1787	440
**Urothelial Cancer**	0.742	−1.346	0.713	−1.322	0.075	1538	14	5.178	3.919	4403	1532	501

### Anticancer metabolic drugs and their targets through centralities

**Figure 3:**
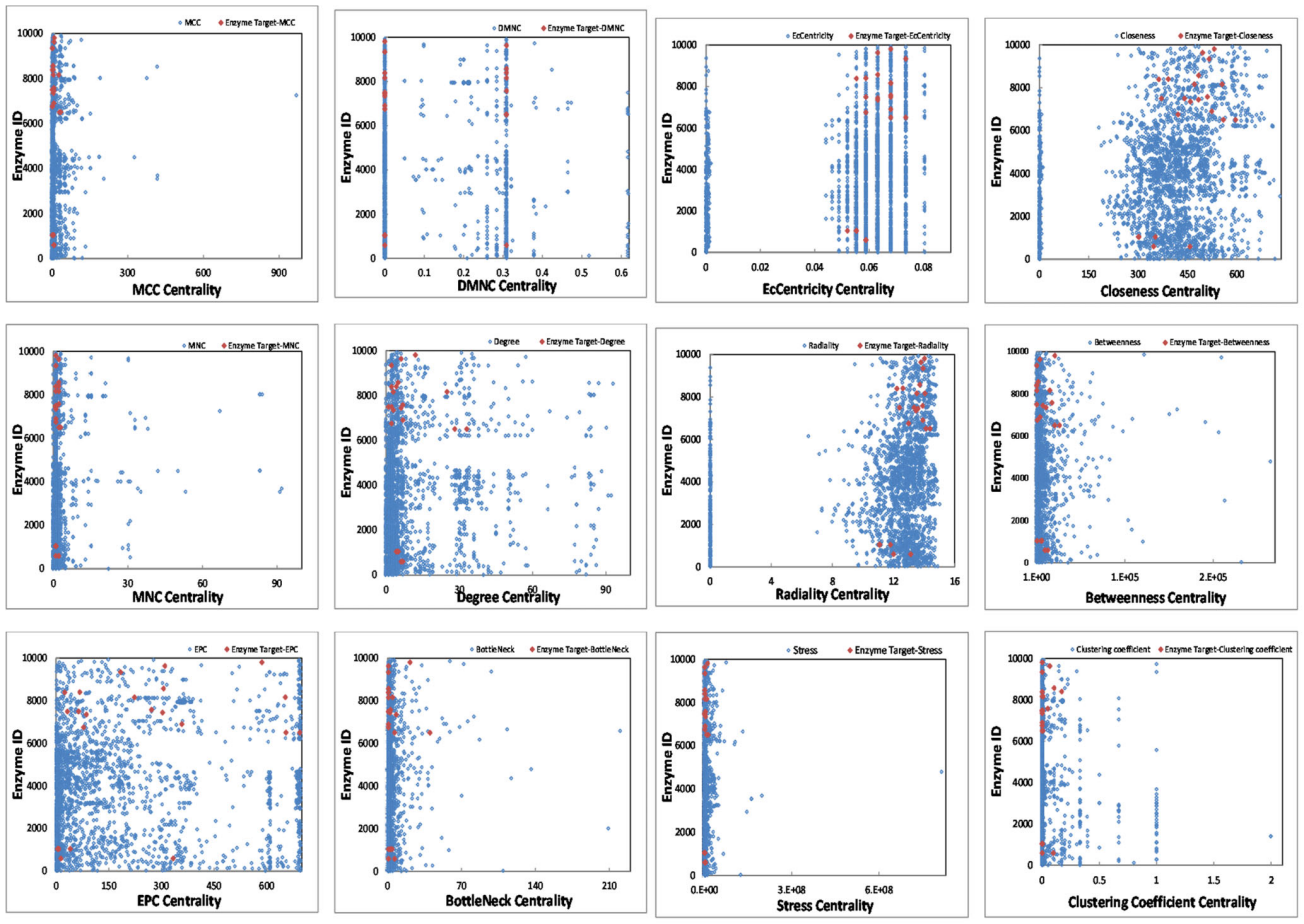
Cluster distribution in enzyme and metabolite-centric cancer and normal networks.

Centrality parameters are global properties of a network that rank graph nodes according to their importance in the network. The higher the rank, the more important a node is in the network, indicating that it may play key roles in controlling cellular functions. We have carried out centrality analysis for directed enzyme-centric networks. All enzymes in 15 enzyme-centric networks have been sorted according to 12 different centrality parameters in order to check whether drug targets appears as highly connected nodes. As Figure 3 shows, drug targets are not available among the 100 top of 12 different centralities. All centrality data are available in the File_S9.

### Motif discovery

Network motifs (one of the important local properties of networks) have served as the building blocks of biological networks from bacteria to mammals, and their function has been experimentally studied in some species such as the transcription network of E. coli [62]. For exploring significant differences between metabolite- and enzyme-centric networks of normal and corresponding cancer cell types, size 3 motif analysis has been carried out for these networks. In metabolite-centric networks there was no difference in motif distribution with size 3 between normal and cancer cell types. In addition, motifs with IDs “38, 46, 142, 166, 174, and 238” have positive Z-Scores for metabolic networks. For enzyme-centric networks, motif distributions in size 3 are different in normal and the corresponding cancer cell types. For example, in the breast enzyme-centric network, the feed forward loop (ID = 38) has a positive Z-Score in breast cancer but a negative Z-Score in the normal cell type (Table 4). All motif data are available in the File_S10.

**Table 4:** Motif finding in directed metabolic and enzyme-centric breast networks.

Motif ID	Enzyme centric network		Metabolic centric network	
	Breast Cancer	Normal - Breast Grandular	Breast Cancer	Normal - Breast Grandular
	Z-SCORE	Z-SCORE	Z-SCORE	Z-SCORE
6	−0.297858	6.904595	−6.640324	−5.26027
14	−14.077722	−5.136464	−14.989574	−16.436516
34	−11.134564	−4.373875	−11.714442	−8.455331
36	−4.061103	2.328047	−6.202353	−4.754256
38	1.447868	−3.813574	5.214613	3.870699
46	15.150644	6.304231	3.585829	3.334919
78	6.237981	11.685102	−12.759671	−8.828319
140	52.125441	36.997489	−3.720983	−3.250312
142	4.300469	4.41293	40.237059	42.413737
164	3.747944	10.054664	−18.118178	−18.989507
166	−3.970751	−5.07765	5.691384	5.705127
174	−2.890623	−3.240451	5.873318	8.981136
238	NA	−11.224703	17.323516	5.615659

### Clustering

**Figure 4:**
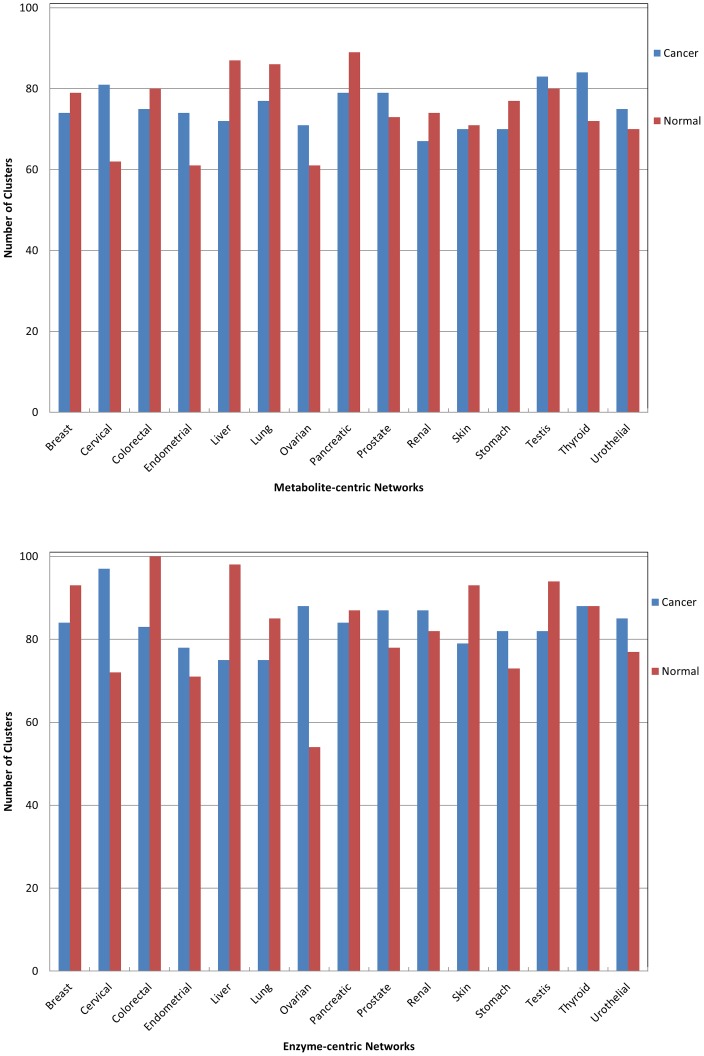
Distribution of drug targets in twelve different centralities.

We have carried out clustering analysis for exploring significant differences in the number of clusters in directed enzyme-centric networks of normal and corresponding cancer cell types. Results related to the *MCODE* clustering algorithm (Figure 4) show that the number of clusters in metabolite-centric networks in both normal and cancer cell types reveal no significant discrepancy. This is also true for the enzyme-centric networks of normal and cancer cell types. All clustering data are available in the File_S11.

### Anticancer metabolic drugs and their targets through clusters

**Figure 5:**
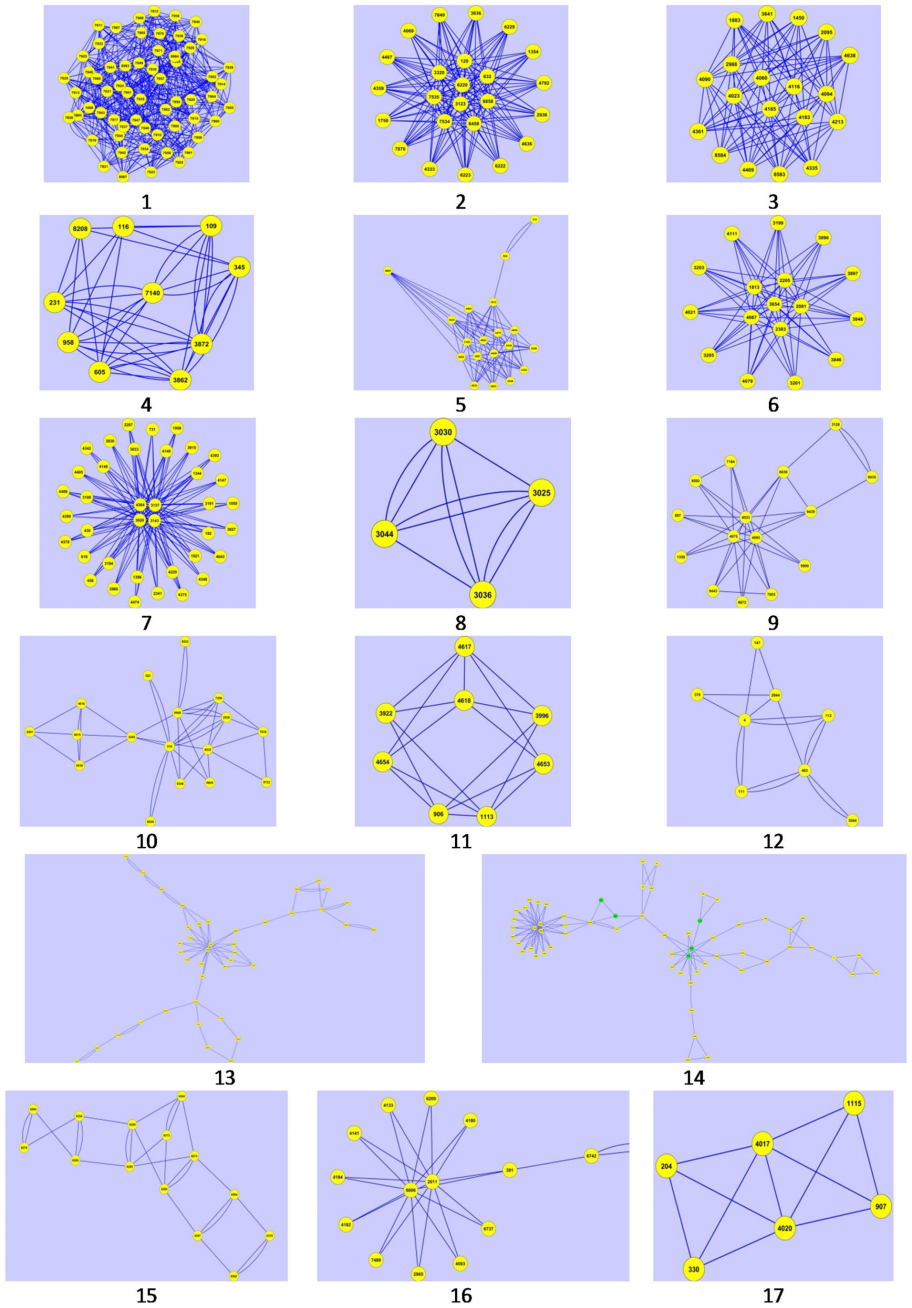
First 17 clusters of liver enzyme-centric cancer network. Drug targets (green nodes) are in cluster number 14.

In the next step, we have identified the drug target of anticancer metabolic drugs (extracted from the drug bank) through clusters in the enzyme-centric network of cancer cell types. The results shows drug targets gather in a specific cluster of an enzyme-centric network of the cancer cell (Figure 5, cluster number 14). All clustering data are available in the File_S11.

## Discussion

Networks are considered to be a major representation of many nonlinear complex systems in the real world. The theory of complex networks uses methods previously developed in graph theory, statistics, physics, and computer simulation in order to study the topological features and relationships between structure and function in the formation of different real world networks such as social, information, and biological networks. Structural network controllability is a new field guiding a system's behavior towards a desired state through appropriate management of some input variables. The difficulty in control theory is because of the fact that two independent factors (with its own layer of unknown) contribute to controllability [48]: (1) the system's architecture, represented by the network in which components interact with each other; and (2) the dynamical rules that capture the time-dependent interactions between the components. Therefore, controllability has been possible just in systems where both issues are well mapped, for instance rate control for communication networks, small biological circuits, and the control of synchronized networks [45], [66], [67]. From the advances towards understanding complex networks accumulated in the last decade, we know that network topology fundamentally affects the dynamical processes on it, from epidemic spreading to synchronization phenomenon. So, it is fair to expect that the network topology would definitely affect controllability as well. This approach helps us avoid any entanglement due to nonlinear effects [49]. In addition, this kind of controllability (structural controllability) allows consideration of networks of arbitrary size (with thousands to millions of nodes). In structural network controllability, both nodal and edge dynamics frameworks have been implied for covering unipartite graphs [48], [50], [54]. In the nodal dynamics approach, Liu's work assumes that only driver node values could be directly controlled through external signals whereas Nacher's study (MDS method) undertakes that each driver node is sufficiently smart to control individual links separately [48], [50]. Although these two works have provided different results especially in targeting highly connected nodes by driver nodes, the authors mention that their results do not contradict because they use different strategies. Thus they believe that the MDS approach complements Liu's results [50].

In this study, we have explored the relationships between structural network controllability, topological parameters, and network medicine (metabolic drug targets). We have applied a topological analysis to genome-scale metabolic models of 15 normal and corresponding cancer cell types. First, we have constructed metabolite- and enzyme-centric networks based on the metabolic SBML files. We have performed primary topological analysis to check whether there are any structural differences in the metabolite-centric and enzyme-centric of normal and cancer metabolic networks. The results show all constructed networks satisfy scale-free and small-world properties. But there is not any significant differences between normal and cancer tissues. Next, we have used the MDS concept in metabolic networks since controlling cancer metabolism through internal signals seems more reasonable biologically. Metabolic networks are appropriate choice because they allow us to consider both metabolite-centric (nodes in original network) and enzyme-centric (edges in original network) networks separately. Based on an assumption (the targets of approved anticancer metabolic drugs are driver nodes and therefore control cancer metabolic networks), we wanted to explore whether it is possible to explore topological parameters which could specify driver nodes in the metabolic networks. So, we have done two studies based on the MDS controllability concept in the enzyme-centric metabolic networks: 1) to check whether driver nodes tend to be part of centrality indexes such as highly connected nodes (Hubs). 2) to explore topological parameters which could specify driver nodes in the metabolic networks.

In performing centrality analysis, the distribution of drug targets among the 100 top of twelve centrality parameters was not significant. It means that drug targets avoid being highly connected enzymes. So, different centralities used in this study could not consider as a driver node for controlling systems. Motifs, as another local property of networks, have also been examined and there was no difference in metabolite-centric networks of cancer and normal cell types, but there were significant discrepancies in the enzyme-centric networks of cancer cells and their corresponding normal cell types. The number of clusters between cancer and corresponding normal cell networks show no significant differences, but characterizing drug targets in enzyme-centric clusters shows that most of the drug targets belong in one specific cluster of an enzyme-centric network. Therefore our results indicate that besides primary network parameters, more complex network metrics such as motifs and clusters may be also appropriate parameters for controlling the metabolic systems. Besides, for metabolic networks, enzyme-centric networks could be more reliable in the context of controllability, although little attention has been paid to such networks in systems controllability. The outcomes of metabolic network controllability could create insights into the discovery of novel drug targets.The results also suggest considering DCS [55], [56] instead of nodal control could lead to a new strategy for cancer treatment in the network medicine field.

## Supporting Information

File S1 **Compare metabolites and reactions between normal and cancer models (including all networks).**
(RAR)

File S2 **Lists of metabolites and reactions of cancers models.**
(XLSX)

File S3 **Lists of metabolites and reactions of normal models.**
(XLSX)

File S4 **Constructed networks (including all normal and cancer networks).**
(RAR)

File S5 **Summary definition of the different parameters.**
(DOC)

File S6 **Anticancer metabolic drugs and their targets.**
(XLSX)

File S7 **Metabolic functions of the drug targets.**
(XLSX)

File S8 **Primary topological parameters for all constructed networks (including metabolite- and enzyme-centric directed and undirected networks).**
(RAR)

File S9 **Centrality data (including all enzyme-centric cancer networks).**
(RAR)

File S10 **Motif data (including metabolite- and enzyme-centric normal and cancer networks).**
(RAR)

File S11 **Clustering data (including all enzyme-centric cancer networks).**
(RAR)

File S12 **Power-law plots for every constructed network with fitting results (including metabolite- and enzyme-centric directed and undirected networks).**
(RAR)

File S13 **Network construction procedures.**
(DOCX)
