# Supplementary material for: Controllability in Cancer Metabolic Networks According to Drug Targets as Driver Nodes
Source: PLoS One. 2013 Nov 25;8(11):e79397. doi: 10.1371/journal.pone.0079397 (PMC3839908; doi:10.1371/journal.pone.0079397)
Supplement: File S5 — Summary definition of the different parameters. (DOC) [file pone.0079397.s005.doc]

**Graph Theory – Some Definitions**

A mathematical representation of a network is a **graph** G(V,E). Its **vertex** set (V) consists of all nodes. Two nodes are adjacent if there is an edge between which connects them.

The clustering coefficient (CC) is a ratio *N* / *M*, where *N* is the edges numbers between the neighbors of *n*, and *M* is the maximum edge numbers which could possibly exist between the neighbors of *n*. The CC value of a node is a number between [0,1].

In undirected networks, the **clustering coefficient *C_n_*** of a node *n* is defined as *C_n_* = 2*e_n_*/(*k_n_*(*k_n_*-1)), where *k_n_* is the number of neighbors of *n* and *e_n_* is the number of connected pairs between all neighbors of *n* .

In directed networks, the definition is slightly different: *C_n_* = *e_n_*/(*k_n_*(*k_n_*-1)).

The **network clustering coefficient** is the average of the clustering coefficients for all nodes in the network. Here, nodes with less than two neighbors are assumed to have a clustering coefficient of 0.

The **number of connected components** indicates the connectivity of a network (a lower number of connected components suggests a stronger connectivity). The length of a path is the number of edges forming it. There may be multiple paths connecting two given nodes.

The **network diameter** is the largest distance between two nodes. If a network is disconnected, its diameter is the maximum of all diameters of its connected components.

The **network radius** is the minimum among the non-zero eccentricites of the nodes in the network.

The shortest path length, also called distance, between two nodes *n* and *m* is denoted by *L*(*n*,*m*).

The **average shortest path length**, also known as the **characteristic path length**, gives the expected distance between two connected nodes.

The neighborhood of a given node *n* is the set of its neighbors. The connectivity of *n*, denoted by *k_n_*, is the size of its neighborhood. The **average number of neighbors** indicates the average connectivity of a node in the network.

A normalized version of this parameter is the **network density**. The density is a value between 0 and 1. It shows how densely the network is populated with edges (self-loops and duplicated edges are ignored). A network which contains no edges and solely isolated nodes has a density of 0. In contrast, the density of a clique is 1.

The **number of isolated nodes** is number of nodes without having any edges.

A **loop** (also called a **self-loop**) is an edge that connects a vertex to itself.

The number of **multi-edge node pairs** indicates how often neighboring nodes are linked by more than one edge.

In undirected networks, the **node degree** of a node *n* is the number of edges linked to *n*. A self-loop of a node is counted like two edges for the node degree. The **node degree distribution** gives the number of nodes with degree *k* for *k = 0,1,…*.

In directed networks, the **in-degree** of a node *n* is the number of incoming edges and the **out-degree** is the number of outgoing edges. Similar to undirected networks, there are an **in-degree distribution** and an **out-degree distribution**.

The **connectivity** of a node is the number of its neighbors. The **neighborhood connectivity** of a node *n* is defined as the average connectivity of all neighbors of *n* . The **neighborhood connectivity distribution** gives the average of the neighborhood connectivities of all nodes *n* with *k* neighbors for *k = 0,1,…*.

The **betweenness centrality** ***C_b_(n)*** of a node *n* is computed as follows:
*C_b_*(*n*) = ∑*_s≠n≠t_* (*σ_st_* (*n*) / *σ_st_*),

where *s* and *t* are nodes different from *n* in the network, *σ_st_* is of shortest paths number from *s* to *t*, and *σ_st_* (*n*) is the shortest paths number from *s* to *t* in which *n* lies on. Betweenness centrality is computed just for networks without containing multiple edges. For each node *n* the betweenness value is normalized by dividing the number of node pairs excluding *n*: (*N*-1)(*N*-2)*/2*, where *N* is the total number of nodes in the connected component that *n* belongs to. Thus, the betweenness centrality of a node is a number between [0, 1].

The **closeness centrality** ***C_c_(n)*** of a node *n* is defined as follows:

*C_c_*(*n*) = *1* / *avg*( *L*(*n*,*m*) )

where *L*(*n*,*m*) is the length of the shortest path between nodes *n* and *m*. The closeness centrality of a node is a number between [0, 1]. Closeness centrality quantitatively measures information transition from a given node to other accessible nodes in the network .

The **stress** of a node *n* is the shortest paths number passing through *n*. If a node contains high shortest paths number it has a high stress. This parameter is defined only for networks without multiple edges. The stress distribution gives the number of nodes with stress *s* for different values of *s*.

As there is no formal definition of what a **hub** is, there exist different definitions for a node to be a hub . But the common agreement is that Hubs are defined as a topological property. Hub definition based on degree and functionality are two useful criteria.

**Network motifs:** basic interaction patterns which repeat throughout biological networks . This occurrence has been seen much more than in random networks. It seems motifs are the building blocks of transcription networks in all organisms.

Milgram found that each two random nodes could connect to each other through on average 5–6 intermediate steps . This suggests that such networks are **small-world**, prompting the popular phrase “six-degrees of separation”. Metabolic networks also show the small-world property

**Clusters** are set of different groups so that elements of one group are more similar to each other than to those in other groups. The task of assigning clusters into various groups is called cluster analysis or clustering. There are a lot of algorithms for Cluster analysis which differ significantly in efficiency and effectiveness of finding them.

**References:**

1. Barabasi, A.L. and Z.N. Oltvai, *Network biology: understanding the cell's functional organization.* Nat Rev Genet, 2004. **5**(2): p. 101-13.

2. Watts, D.J. and S.H. Strogatz, *Collective dynamics of 'small-world' networks.* Nature, 1998. **393**(6684): p. 440-2.

3. Maslov, S. and K. Sneppen, *Specificity and stability in topology of protein networks.* Science, 2002. **296**(5569): p. 910-3.

4. Brandes, U., *A Faster Algorithm for Betweenness Centrality.* Journal of Mathematical Sociology, 2001. **25**: p. 163-177.

5. Newman, M.E.J., *A measure of betweenness centrality based on random walks.* Soc Networks, 2005. **27**(1): p. 16-16.

6. Shimbel, A., *Structural parameters of communication networks.* Bulletin of Mathematical Biology, 1953. **15**(4): p. 501-507.

7. Sporns, O., C.J. Honey, and R. Kotter, *Identification and classification of hubs in brain networks.* PLoS One, 2007. **2**(10): p. e1049.

8. Alon, U., *Network motifs: theory and experimental approaches.* Nat Rev Genet, 2007. **8**(6): p. 450-61.

9. Milgram, S., *The Small World Problem.* Psychology Today, 1967. **2**: p. 60-67.

10. Jeong, H., et al., *The large-scale organization of metabolic networks.* Nature, 2000. **407**(6804): p. 651-4.

11. Wagner, A. and D.A. Fell, *The small world inside large metabolic networks.* Proc Biol Sci, 2001. **268**(1478): p. 1803-10.
