# Supplementary material for: Controllability in Cancer Metabolic Networks According to Drug Targets as Driver Nodes
Source: PLoS One. 2013 Nov 25;8(11):e79397. doi: 10.1371/journal.pone.0079397 (PMC3839908; doi:10.1371/journal.pone.0079397)
Supplement: File S13 — Network construction procedures. (DOCX) [file pone.0079397.s013.docx]

**Construction Metabolite- and Enzyme-Centric Networks**

*Definition*: In a metabolite-centric network, metabolites are nodes and a link denotes the involvement of any two metabolites used by a given enzyme.

- **Construction an metabolite-centric network:**

Using a SBML file as input, an undirected metabolite-centric network has been constructed by dot product of binary stoichiometric matrix and the transpose of binary stoichiometric matrix. For construction a directed metabolite-centric network, each column of the stoichiometric matrix has been parsed and added an edge per every value sign changes (positive to negative or vice versa). It also considers the reversibility of reactions.

*Note:* single nodes have also been considered in the construction process.

**Algorithm of Undirected Metabolite-Centric Network Construction**

**(In Matlab Programming Format)**

-----------------------------------------------------------------------------------------------------------------------

model=readCbModel(fileName);

% construction of Stoichiometric Matrix (comma separated Format)

fout = fopen('Stoich_Matrix.txt', 'w+');

dlmwrite(fout,full(model.S));

% construction of Binary Stoichiometric Matrix (comma separated Format)

S_bin=zeros(size(model.S));

S_bin(find(model.S))=1;

fout2 = fopen('Binary_Stoich_Matrix.txt', 'w+');

dlmwrite(fout2,full(S_bin));

% construction of Undirected-Metabolite-Metabolite Network (comma separated Format)

Acomp=S_bin*S_bin';

fout3 = fopen('Metabolite_Cent_csv.txt', 'w+');

dlmwrite(fout3,full(Acomp));

% reading a Metabolic-Metabolic comma separated file

% Select "first column" as "Source Interaction" and "second column" as "Target Interaction"

s = csvread(fout3);

[m,n]=size(s);

fout4 = fopen('Undir_Metabolite_Cent.txt', 'w+');

for row=1:m

num=0;

% because cell(i,j)=cell(j,i) we must delete duplicate entries by putting

% col=row:n in the second if command. since we must ignore diagonal elements,

% the counter will be col=row+1:n

for col=row+1:n

% edge are those which includes number not equal to zero

if s(row,col)~=0

fprintf(fout4,'%s\t%s\t%d\n',model.mets{row},model.mets{col},s(row,col));

num=num+1;

end

end

% considering nodes which do not contain any edges

if num==0

fprintf(fout4,'%s\n',model.mets{row});

end

end

fclose(fout);

fclose(fout2);

fclose(fout3);

fclose(fout4);

% End of program

-----------------------------------------------------------------------------------------------------------------------

**Algorithm of Directed Metabolite-Centric Network Construction**

**(In Matlab Programming Format)**

-----------------------------------------------------------------------------------------------------------------------

fid = fopen('carr_mets.txt');

tline = fgetl(fid);

i=1;

Curr_met={};

while ischar(tline)

Curr_met{i,1}=tline;

tline = fgetl(fid);

i=i+1;

end

fclose(fid);

[h,g]=size(Curr_met);

model=readCbModel(fileName);

[m,n]=size(model.S);

fout = fopen(' Dir_Metabolite_Cent.txt', 'w+');

% finds non-zero elements of the S-matrix (in order to make the algorithm faster),

% parses through each column, and considers an edge for every unlike-signs.

% It also consider Reversibility for Enzyme-Enzyme network.

num=zeros(size(model.mets));

for j=1:n

indices=find(model.S(:,j));

[a,b]=size(indices);

r=0;

if a~=0

r=1;

end

while r<a

i=1;

while i<(a-r+1)

N=0;

for k=1:h

if strcmp(model.metNames{indices(r,1)},Curr_met{k,1})==1

N=1;

break

elseif strcmp(model.metNames{indices(r+i,1)},Curr_met{k,1})==1

N=1;

break

end

end

if model.S(indices(r,1),j)<0 && model.S(indices(r+i,1),j)>0

if model.rev(j)==1 && N==0

fprintf(fout,'%s\t%s\t%s\n',model.mets{indices(r,1)},'reaction-product',model.mets{indices(r+i,1)});

fprintf(fout,'%s\t%s\t%s\n',model.mets{indices(r+i,1)},'reaction-product',model.mets{indices(r,1)});

else

fprintf(fout,'%s\t%s\t%s\n',model.mets{indices(r,1)},'reaction-product',model.mets{indices(r+i,1)});

end

elseif model.S(indices(r,1),j)>0 && model.S(indices(r+i,1),j)<0

if model.rev(j)==1 && N==0

fprintf(fout,'%s\t%s\t%s\n',model.mets{indices(r+i,1)},'reaction-product',model.mets{indices(r,1)});

fprintf(fout,'%s\t%s\t%s\n',model.mets{indices(r,1)},'reaction-product',model.mets{indices(r+i,1)});

else

fprintf(fout,'%s\t%s\t%s\n',model.mets{indices(r+i,1)},'reaction-product',model.mets{indices(r,1)});

end

end

i=i+1;

end

r=r+1;

end

end

% considering nodes which do not contain any edges

for k=1:m

if num(k,1)==0

fprintf(fout,'%s\n',model.mets{k});

end

end

fclose(fout);

% End of program

-----------------------------------------------------------------------------------------------------------------------

*Definition*: In an enzyme-centric network, enzymes are nodes and the enzymes are connected if the products of the reaction represented by one enzyme become the substrate of another enzyme.

- **Construction an enzyme-centric network:**

Using a SBML file as input, an undirected enzyme-centric network has been constructed by dot product of the transpose of binary stoichiometric matrix and binary stoichiometric matrix. For construction a directed enzyme-centric network, each row of the stoichiometric matrix has been parsed and added an edge per every value sign changes (positive to negative or vice versa). It also considers the reversibility of reactions.

*Note:* providing a given input text file including currency metabolites, the algorithm removes those metabolites and builds an enzyme-centric network using the new stoichiometry matrix.

*Note:* single nodes have also been considered in the construction process.

**Algorithm of Undirected Enzyme-Centric Network Construction**

**(In Matlab Programming Format)**

-----------------------------------------------------------------------------------------------------------------------

model=readCbModel(fileName);

% construction of Stoichiometric Matrix (comma separated Format)

fout = fopen('Stoich_Matrix_csv.txt', 'w+');

dlmwrite(fout,full(model.S));

% construction of Binary Stoichiometric Matrix (comma separated Format)

S_bin=zeros(size(model.S));

S_bin(find(model.S))=1;

fout2 = fopen('Binary_Stoich_Matrix.txt', 'w+');

dlmwrite(fout2,full(S_bin));

% construction of Undirected-Enzyme-Enzyme Network (comma separated Format)

Aenz=S_bin'*S_bin;

fout3 = fopen('Enzyme_Cent.txt', 'w+');

dlmwrite(fout3,full(Aenz));

% Select "first column" as "Source Interaction" and "second column" as "Target Interaction"

ss = csvread(outname3);

[g,h]=size(ss);

fout4 = fopen('Enzyme_Cent_single_node.txt', 'w+');

fout = fopen(fout4, 'w+');

for row=1:g

num=0;

% because cell(i,j)=cell(j,i) we must delete duplicate entries by putting

% col=row:h in the second if command. since we must ignor diagonal elements,

% the counter will be col=row+1:h

for col=row+1:h

% edge are those which includes number not equal to zero

if ss(row,col)~=0

fprintf(fout,'%s\t%s\t%d\n',model.rxns{row},model.rxns{col},ss(row,col));

num=num+1;

end

end

% considering nodes which do not contain any edges

if num==0

fprintf(fout,'%s\n',model.rxns{row});

end

end

fclose(fout);

fclose(fout2);

fclose(fout3);

fclose(fout4);

% End of program

-----------------------------------------------------------------------------------------------------------------------

**Algorithm of Directed Enzyme-Centric Network Construction**

**(In Matlab Programming Format)**

-----------------------------------------------------------------------------------------------------------------------

% reading the Library text file and construct array of currency metabolites

fid = fopen(fileName1);

tline = fgetl(fid);

i=1;

Curr_met={};

while ischar(tline)

Curr_met{i,1}=tline;

tline = fgetl(fid);

i=i+1;

end

fclose(fid);

[h,g]=size(Curr_met);

model=readCbModel(fileName2);

[m,n]=size(model.S);

% reading the Metabolites array and check their availability in the library text file

N_curr=zeros(m,1);

for q=1:m

for i=1:h

if strcmp(model.metNames{q},Curr_met{i,1})==1

N_curr(q,1)=N_curr(q,1)+1;

end

end

end

% Remove metabolites which are in the input Currecny Metabolites list

for q=1:m

if N_curr(q,1)~=0

for i=1:n

model.S(q,i)=0;

end

end

end

fout = fopen('Dir_Enzyme_Cent.txt', 'w+');

% finds non-zero elements of the S-matrix (in order to make the algorithm faster),

% parses through each row, and considers an edge for every unlike-signs.

% It also consider Reversibility for Enzyme-Enzyme network.

num=zeros(size(model.rxns));

for j=1:m

indices=find(model.S(j,:));

[a,b]=size(indices);

r=0;

if b~=0

r=1;

end

while r<b

i=1;

while i<(b-r+1)

if model.S(j,indices(1,r))>0 && model.S(j,indices(1,r+i))<0

if model.rev(indices(r+i))==1 && model.rev(indices(r))==1

fprintf(fout,'%s\t%s\t%s\n',model.rxns{indices(1,r)},'reaction-product',model.rxns{indices(1,r+i)});

fprintf(fout,'%s\t%s\t%s\n',model.rxns{indices(1,r+i)},'reaction-product',model.rxns{indices(1,r)});

num(1,indices(1,r))=1;

num(1,indices(1,r+i))=1;

else

fprintf(fout,'%s\t%s\t%s\n',model.rxns{indices(1,r)},'reaction-product',model.rxns{indices(1,r+i)});

num(1,indices(1,r))=1;

num(1,indices(1,r+i))=1;

end

elseif model.S(j,indices(1,r))<0 && model.S(j,indices(1,r+i))>0

if model.rev(indices(r+i))==1 && model.rev(indices(r))==1

fprintf(fout,'%s\t%s\t%s\n',model.rxns{indices(1,r+i)},'reaction-product',model.rxns{indices(1,r)});

fprintf(fout,'%s\t%s\t%s\n',model.rxns{indices(1,r)},'reaction-product',model.rxns{indices(1,r+i)});

num(1,indices(1,r))=1;

num(1,indices(1,r+i))=1;

else

fprintf(fout,'%s\t%s\t%s\n',model.rxns{indices(1,r+i)},'reaction-product',model.rxns{indices(1,r)});

num(1,indices(1,r))=1;

num(1,indices(1,r+i))=1;

end

end

i=i+1;

end

r=r+1;

end

end

% considering nodes which do not contain any edges

for k=1:n

if num(1,k)==0

fprintf(fout,'%s\n',model.rxns{k});

end

end

fclose(fout);

% End of program

-----------------------------------------------------------------------------------------------------------------------
